# Supplementary material for: Criteria and Non-Criteria Antiphospholipid Antibodies in Antiphospholipid Syndrome: How Strong Are They Correlated?
Source: Biomedicines. 2023 Aug 3;11(8):2192. doi: 10.3390/biomedicines11082192 (PMC10452164; doi:10.3390/biomedicines11082192)
Supplement: Supplementary file 1 [file biomedicines-11-02192-s001.zip › biomedicines-2508085-supplementary.pdf]

Supplementary Materials

Supplementary Table S1. Results of ROC analysis evaluating the utility of criteria and non-criteria aPLs in predicting the APS subtype – primary or secondary.

aPLs	Area Under the Curve	95% CI	
		Lower limit	Upper limit
aCL IgG	0.348	0.216	0.480
aCL IgM	0.440	0.303	0.577
aβ2GPI IgG	0.500	0.357	0.642
aβ2GPI IgM	0.447	0.311	0.583
aPE IgG	0.503	0.363	0.644
aPE IgM	0.493	0.349	0.637
aPS IgG	0.399	0.265	0.534
aPS IgM	0.427	0.290	0.564
aPT IgG	0.517	0.377	0.658
aPT IgM	0.444	0.301	0.587

APS – antiphospholipid syndrome; aPLs – antiphospholipid antibodies; aCL – anti-cardiolipin antibodies; aβ2GPI – anti-β2 glycoprotein I antibodies; aPE – anti-phosphatidylethanolamine antibodies; aPS – anti-phosphatidylserine antibodies; aPT – anti-prothrombin antibodies; Ig – immunoglobulin; ROC – receiver operating characteristic curve.

Supplementary Table S2. Results of ROC analysis evaluating the utility of criteria and non-criteria aPLs in predicting deep venous thrombosis, deep venous thrombosis associated with arterial thrombosis, and deep venous thrombosis associated with obstetric morbidity.

aPLs	Deep Venous Thrombosis			Deep Venous Thrombosis + Arterial Thrombosis			Deep Venous Thrombosis + Obstetric Morbidity		
	Area Under the Curve	95% CI		Area Under the Curve	95% CI		Area Under the Curve	95% CI	
		Lower limit	Upper limit		Lower limit	Upper limit		Lower limit	Upper limit
aCL IgG	0.411	0.276	0.547	0.339	0.110	0.567	0.487	0.148	0.826
aCL IgM	0.422	0.284	0.560	0.388	0.102	0.674	0.544	0.123	0.964
aβ2GPI IgG	0.429	0.288	0.570	0.388	0.162	0.614	0.614	0.285	0.942
aβ2GPI IgM	0.372	0.234	0.509	0.445	0.209	0.682	0.602	0.237	0.968
aPE IgG	0.350	0.213	0.487	0.165	0.064	0.267	0.348	0.057	0.640
aPE IgM	0.387	0.253	0.521	0.307	0.146	0.469	0.462	0.103	0.822
aPS IgG	0.463	0.326	0.600	0.341	0.099	0.584	0.502	0.161	0.843
aPS IgM	0.442	0.303	0.581	0.350	0.100	0.600	0.559	0.158	0.959
aPT IgG	0.335	0.199	0.470	0.355	0.139	0.572	0.506	0.201	0.811
aPT IgM	0.422	0.285	0.559	0.368	0.144	0.593	0.667	0.401	0.933

aPLs – antiphospholipid antibodies; aCL – anti-cardiolipin antibodies; aβ2GPI – anti-β2 glycoprotein I antibodies; aPE – anti-phosphatidylethanolamine antibodies; aPS – anti-phosphatidylserine antibodies; aPT – anti-prothrombin antibodies; Ig – immunoglobulin; ROC – receiver operating characteristic curve.

Supplementary Table S3. Results of ROC analysis evaluating the utility of criteria and non-criteria aPLs in predicting arterial thrombosis, arterial thrombosis associated with obstetric morbidity, and arterial thrombosis associated with deep venous thrombosis and obstetric morbidity.

aPLs	Arterial Thrombosis			Arterial Thrombosis + Obstetric Morbidity			Arterial Thrombosis + Deep Venous Thrombosis + Obstetric Morbidity		
	Area Under the Curve	95% CI		Area Under the Curve	95% CI		Area Under the Curve	95% CI	
		Lower limit	Upper limit		Lower limit	Upper limit		Lower limit	Upper limit
aCL IgG	0.462	0.316	0.608	0.392	0.103	0.681	0.533	0.126	0.940
aCL IgM	0.468	0.325	0.612	0.595	0.280	0.909	0.533	0.000	1.000
aβ2GPI IgG	0.455	0.311	0.599	0.409	0.074	0.744	0.728	0.475	0.980
aβ2GPI IgM	0.525	0.382	0.668	0.706	0.572	0.841	0.706	0.497	0.915
aPE IgG	0.473	0.332	0.614	0.453	0.181	0.724	0.221	0.069	0.372
aPE IgM	0.415	0.274	0.556	0.396	0.198	0.594	0.426	0.196	0.657
aPS IgG	0.386	0.248	0.524	0.407	0.113	0.701	0.544	0.130	0.958
aPS IgM	0.394	0.255	0.534	0.388	0.030	0.747	0.651	0.172	1.000
aPT IgG	0.504	0.369	0.639	0.517	0.274	0.760	0.401	0.055	0.747
aPT IgM	0.430	0.285	0.575	0.536	0.234	0.838	0.599	0.166	1.000

aPLs – antiphospholipid antibodies; aCL – anti-cardiolipin antibodies; aβ2GPI – anti-β2 glycoprotein I antibodies; aPE – anti-phosphatidylethanolamine antibodies; aPS – anti-phosphatidylserine antibodies; aPT – anti-prothrombin antibodies; Ig – immunoglobulin; ROC – receiver operating characteristic curve.

Supplementary Table S4. Results of ROC analysis evaluating the utility of criteria and non-criteria aPLs in predicting obstetric morbidity.

aPLs	Area Under the Curve	95% CI	
		Lower limit	Upper limit
aCL IgG	0.488	0.324	0.652
aCL IgM	0.598	0.424	0.772
aβ2GPI IgG	0.510	0.338	0.681
aβ2GPI IgM	0.650	0.501	0.800
aPE IgG	0.562	0.409	0.714
aPE IgM	0.583	0.414	0.753
aPS IgG	0.573	0.388	0.758
aPS IgM	0.531	0.338	0.724
aPT IgG	0.631	0.448	0.778
aPT IgM	0.587	0.442	0.732

aPLs – antiphospholipid antibodies; aCL – anti-cardiolipin antibodies; aβ2GPI – anti-β2 glycoprotein I antibodies; aPE – anti-phosphatidylethanolamine antibodies; aPS – anti-phosphatidylserine antibodies; aPT – anti-prothrombin antibodies; Ig – immunoglobulin; ROC – receiver operating characteristic curve.

Supplementary Table S5. Results of ROC analysis evaluating the utility of criteria and non-criteria aPLs in predicting the non-thrombotic clinical manifestations of APS.

aPLs	Area Under the Curve	95% CI	
		Lower limit	Upper limit
aCL IgG	0.493	0.344	0.641
aCL IgM	0.421	0.285	0.558
aβ2GPI IgG	0.405	0.269	0.542
aβ2GPI IgM	0.365	0.234	0.495
aPE IgG	0.569	0.431	0.707
aPE IgM	0.479	0.335	0.622
aPS IgG	0.532	0.391	0.674
aPS IgM	0.514	0.375	0.653
aPT IgG	0.469	0.329	0.610
aPT IgM	0.366	0.226	0.506

APS – antiphospholipid syndrome; aPLs – antiphospholipid antibodies; aCL – anti-cardiolipin antibodies; aβ2GPI – anti-β2 glycoprotein I antibodies; aPE – anti-phosphatidylethanolamine antibodies; aPS – anti-phosphatidylserine antibodies; aPT – anti-prothrombin antibodies; Ig – immunoglobulin; ROC – receiver operating characteristic curve.
